# Complete Genome Sequence of Human Norovirus Strain GII.P7-GII.6 Detected in a Patient in the United States in 2014

**DOI:** 10.1128/genomeA.01211-16

**Published:** 2016-10-20

**Authors:** Zhihui Yang, Jan Vinjé, Christopher A. Elkins, Michael Kulka

**Affiliations:** a Division of Molecular Biology, Office of Applied Research and Safety Assessment, Center for Food Safety and Applied Nutrition, U.S. Food and Drug Administration, Laurel, Maryland, USA; b Division of Viral Diseases, Centers for Disease Control and Prevention, Atlanta, Georgia, USA

## Abstract

Homologous recombination is one of the driving forces contributing to the genetic variation of human norovirus, which is an important cause of sporadic and epidemic acute gastroenteritis globally. We report the near-complete genome of the novel recombinant norovirus strain GII.P7-GII.6, detected in an adult with norovirus gastroenteritis in the United States.

## GENOME ANNOUNCEMENT

Since the discovery and identification of Norwalk virus in 1972 (1), human noroviruses have been identified as the leading cause of acute viral gastroenteritis worldwide (2). This genetically diverse group of viruses form a separate genus in the family *Caliciviridae* that cluster phylogenetically into at least seven genogroups with more than 30 genotypes located within three human-containing genogroups (I, II, and IV) (3). Based on the sequence data to date, the majority of nucleotide differences between and within genotypes/strains is reported at the level of individual nucleotide changes and may be driven by a combination of selection pressure(s) and the generally lower fidelity of RNA-dependent RNA polymerases (4). In contrast, genome recombination is an alternative mechanism contributing to the genetic diversity of norovirus and typically occurs within or close to the junction of open reading frames 1 (ORF1) and 2 (ORF2) (5, 6). In the 2014–2015 season, several GII.P7-GII.6 strains were identified from both sporadic cases and as the cause of large outbreaks in Europe and Asia (GenBank accession no. KM924012, KM036376, and LN854568). During the same period, GII.6 strains, typed based on partial ORF2 sequences, accounted for 11% of the 893 norovirus outbreaks reported to CaliciNet (http://www.cdc.gov/norovirus/reporting/calicinet/data.html). Until routine dual genotyping (partial polymerase and capsid sequenced) is conducted, norovirus recombinant viruses cannot be routinely identified. Here, we report the first near-complete genome sequence of a norovirus GII.P7-GII.6 strain associated with a sporadic case of acute gastroenteritis in the United States.

Viral RNA was extracted from the supernatant of a 10% (wt/vol) norovirus-positive stool sample in phosphate-buffered saline using a QIAampViral RNA minikit (Qiagen). Following library construction using a TruSeq stranded mRNA prep kit (Illumina) and sequencing on the MiSeq platform (Illumina), data were analyzed using CLC Genomics Workbench (CLC bio). One contig covering the norovirus genome sequence was assembled from 1,012,519 reads containing an average coverage at 12,203×. The genome sequence of GII.P7-GII.6/Maryland/2014/USA was 7,537 nucleotides (nt) in length, containing (i) three ORFs: ORF1 (near complete), ORF2, and ORF3 of 5,090, 1,644, and 777 nt in length, respectively; and (ii) a 3′ untranslated region of 47 nt in length. Virus typing by using the Norovirus Genotyping tool version 1.0 (7) demonstrated that this sequence was a recombinant strain composed of GII.7 ORF1 and GII.6 ORF2 (bootstrap value = 100). The ORF1 and ORF2 sequences were submitted to BLAST separately and subclustered most closely with GII.P7-GII.6 2014 variants detected in China and Taiwan and the Netherlands with nucleotide differences of 1 to 3% and 1 to 2%, respectively. A full-length genomic sequence BLAST search revealed 97% identity with a GII.P7-GII.6 variant from the Netherlands (GenBank accession no. LN854568). These results indicate that closely related GII.P7-GII.6 variants were circulating worldwide in 2014–2015. Thus, determining the sequence of GII.P7-GII.6/Maryland/2014/USA provides an additional reference sequence for phylogenetic analysis and epidemiological studies regarding circulation of this strain in the United States, particularly with regard to its potential link to outbreaks of foodborne illness. Currently, routine norovirus genotyping by CaliciNet laboratories is being adapted to allow routine dual genotyping of norovirus outbreaks in the United States.

### Accession number(s).

The genome sequence of strain GII.P7-GII.6/Maryland/2014/USA has been deposited in GenBank under the accession number KX268709.

## References

[1] Kapikian AZ, Wyatt RG, Dolin R, Thornhill TS, Kalica AR, Chanock RM 1972 Visualization by immune electron microscopy of a 27-nm particle associated with acute infectious nonbacterial gastroenteritis. J Virol 10:1075–1081. 4117963 10.1128/jvi.10.5.1075-1081.1972 PMC356579

[2] Lopman B, Zambon M, Brown DW 2008 The evolution of norovirus, the “gastric flu”. PLoS Med 5:e01211-16. doi:10.1371/journal.pmed.0050042. PMC2235896 18271623

[3] Zheng DP, Ando T, Fankhauser RL, Beard RS, Glass RI, Monroe SS 2006 Norovirus classification and proposed strain nomenclature. Virology 346:312–323. doi:10.1016/j.virol.2005.11.015. 16343580

[4] Campagnola G, McDonald S, Beaucourt S, Vignuzzi M, Peersen OB 2015 Structure-function relationships underlying the replication fidelity of viral RNA-dependent RNA polymerases. J Virol 89:275–286. doi:10.1128/JVI.01574-14. 25320316 PMC4301111

[5] Bull RA, Tanaka MM, White PA 2007 Norovirus recombination. J Gen Virol 88:3347–3359. doi:10.1099/vir.0.83321-0. 18024905

[6] Worobey M, Holmes EC 1999 Evolutionary aspects of recombination in RNA viruses. J Gen Virol 80:2535–2543. doi:10.1099/0022-1317-80-10-2535. 10573145

[7] Kroneman A, Vennema H, Deforche K, Avoort HVD, Peñaranda S, Oberste MS, Vinjé J, Koopmans M 2011 An automated genotyping tool for enteroviruses and noroviruses. J Clin Virol 51:121–125. doi:10.1016/j.jcv.2011.03.006. 21514213

